# Tooth Loss in Individuals with Dementia: A Swedish Register-Based Cohort Study

**DOI:** 10.1177/00220345251384633

**Published:** 2025-10-22

**Authors:** M. Mohammadi, J. Holmer, H. Imberg, H. Albrektsson, M. Eriksdotter, K. Buhlin

**Affiliations:** 1 Division of Periodontology, Department of Dental Medicine, Karolinska Institutet, Huddinge, Sweden; 2 Department of Periodontology, Specialist Dental Clinic, Västmanland Hospital Västerås, Region Västmanland, Västerås, Sweden; 3 Department of Molecular and Clinical Medicine, Institute of Medicine, Sahlgrenska Academy, University of Gothenburg, Gothenburg, Sweden; 4 Statistiska Konsultgruppen Sweden, Gothenburg, Sweden; 5 Division of Clinical Geriatrics, Centre for Alzheimer Research, Department of Neurobiology, Care Sciences and Society, Karolinska Institutet, Stockholm, Sweden; 6 Inflammation and Aging Theme, Karolinska University Hospital, Stockholm, Sweden; 7 Department of Oral and Maxillofacial Diseases, University of Helsinki, Helsinki, Finland; 8 Public Dental Health Office in Stockholm County, Sweden

**Keywords:** Alzheimer disease, cognitive dysfunction, oral health, cognitive decline, prognostic factor, mortality

## Abstract

Risk factors for dementia include cardiovascular disease, smoking, and diabetes, which also are linked to compromised oral health and periodontal disease. Tooth loss, the hallmark of compromised oral health, is of interest for its systemic effects, including potential impacts on cognitive function. To evaluate tooth loss as a prognostic indicator in dementia, we conducted a register-based cohort study to assess associations of compromised oral health, defined by tooth loss, with mortality risk and progression of cognitive decline. The study population, obtained from linked Swedish nationwide health and quality assurance registries, comprised 3,361 individuals diagnosed with dementia from 2010 to 2013, with follow-up until 2018. Participants were categorized by tooth count: severe tooth loss (<10 remaining teeth), moderate tooth loss (10 to 19 remaining teeth), and a reference group with ≥20 remaining teeth. Mortality rate was analysed by Cox and Poisson regression models, and cognitive decline was assessed by longitudinal analyses of Mini Mental State Examination scores. Analyses were adjusted for demographic and health variables. Tooth loss at the time of dementia diagnosis was not independently associated with increased mortality after covariate adjustment (hazard ratio, 1.12 [95% CI, 0.97 to 1.28] for severe tooth loss vs reference). Annual Mini Mental State Examination scores declined across all groups, with no statistically significant differences among groups. After robust covariate control, no association was observed between tooth loss and increased mortality or cognitive decline in individuals newly diagnosed with dementia. Further studies are needed to determine whether tooth loss is an independent risk factor or a contributing marker in dementia prognosis.

## Introduction

As global life expectancy rises, dementia cases are expected to increase, with 1 in 5 people projected to be aged ≥60 y by 2050 (Prince et al. 2013; “Ageing and Health” 2022). Dementia, affecting 5% to 7% of those aged ≥60y, is a neurodegenerative disorder that impairs cognitive abilities, including memory, language, space-time perception, and problem solving (Prince et al. 2013; Greenberg and Seeley 2022). It remains progressive and fatal, posing significant personal and societal burdens (Livingston et al. 2017). Risk factors include cardiovascular disease, smoking, and diabetes, which also are linked to oral diseases, such as periodontal disease (Genco and Borgnakke 2013; Livingston et al. 2017; Liccardo et al. 2019; Seeley et al. 2022). Previous dementia research has focused mainly on etiologic risk factors. In advanced dementia, prognostic indicators for mortality include older age, poor baseline cognitive function, and poor oral intake (Ramsey and Arnold 2022). Further evidence regarding prognostication in early dementia remains elusive (Melis et al. 2019), and effective treatments are lacking (Gale et al. 2018). Existing therapies such as cholinesterase inhibitors and memantine target symptom relief (Xu et al. 2021; Livingston et al. 2024). Consequently, there is a pressing need for new treatment and management strategies.

Oral health, particularly involving teeth and periodontal disease, has long been studied for its systemic effects, as far back as the late 19th century (Miller 1891). Tooth loss, mainly from caries and periodontal disease, can reduce chewing function and alter dietary habits (Okamoto et al. 2019; Broers et al. 2022). Animal studies show that reduced chewing, as a mechanistic pathway, negatively affects cognitive ability, potentially causing hippocampal neuron loss and impaired synaptogenesis because of reduced somatosensory output from the oral cavity (Kato et al. 1997; Yamamoto and Hirayama 2001; Tsutsui et al. 2007). Tooth loss also may lead to nutritional deficiency and social isolation, which are both risk factors for dementia (Okamoto et al. 2019; Takahashi et al. 2023; Qi et al. 2023; Livingston et al. 2024).

While dementia lacks effective treatments, oral health is highly modifiable, and a positive association between oral status and cognitive ability has been suggested from animal studies (Kato et al. 1997; Tsutsui et al. 2007) and comprehensive epidemiologic studies (Chen et al. 2017; Lee et al. 2020; Ma et al. 2022). Previous research has demonstrated the association of tooth loss with increased mortality risk, potentially due to factors such as impaired mastication, nutritional deficiencies, systemic inflammation, socioeconomic status, and comorbidities (Fang et al. 2018). Moreover, tooth loss has been discussed as a potential prognostic indicator for cognitive decline and dementia (Fang et al. 2018). Given the projected worldwide increase in dementia cases, a better understanding of this association may aid in the search for new approaches to treatment and management of dementia.

The aim of this study was to explore tooth loss as a prognostic indicator for dementia. We hypothesized that compromised oral health, as defined by tooth loss, would be associated with increased risk for mortality and progression of cognitive decline.

## Material and Methods

### Study Design and Setting

To explore tooth loss as a prognostic indicator for dementia in this register-based cohort study, we used data from linked Swedish nationwide health and quality assurance registries. The Swedish National Board of Health and Welfare managed the merger of the data for the registries, which were retrieved pseudonymized. Ethical approval was granted by the Regional Ethical Review Board in Stockholm, Sweden (registration 2017/737-31). This study was reported in accordance with the RECORD guidelines (Reporting of Studies Conducted Using Observational Routinely-Collected Data; Benchimol et al. 2015).

### Data Registries

The Swedish Quality Registry for Caries and Periodontal Diseases (SKaPa) gathers dental records automatically from electronic patient charts by using personal identity numbers (von Bültzingslöwen et al. 2019). All public dental care clinics and the largest private dental care clinics in Sweden are connected to the SKaPa and provide diagnosis and treatment codes reported during the dental care visit. The aim of the Swedish Registry for Cognitive/Dementia Disorders (SveDem) is to improve the quality of diagnostics, treatment, and care for individuals with dementia. Initiated in 2007, this national registry comprises comprehensive data on dementia diagnoses from primary, specialist, and residential care according to *ICD-10* criteria with annual follow-ups (Religa et al. 2015). The National Prescribed Drug Register and the National Patient Register are public authority registries, gathering extensive information about medications, diseases, and treatments (Ludvigsson et al. 2011). Socioeconomic data were collected through the Longitudinal Integrated Database for Health Insurance and Labour Market Studies, another public authority registry (Ludvigsson et al. 2019).

### Study Population

The study population was defined by merging the National Patient Register, National Prescribed Drug Register, SKaPa, and SveDem. This merger allowed us to gather data on oral health, dementia diagnoses, comorbidities, and medications. The study cohort comprised all individuals diagnosed with dementia who had a record in the SKaPa in the same year as the dementia diagnosis or earlier, with an index date of January 1, 2010, and follow-up until December 31, 2018. Participants were included in the study at the point of dementia diagnosis, defined as the date of first prescription for antidementia medication or initial entry into the SveDem between January 1, 2010, and December 31, 2013, resulting in 3,361 eligible participants. Follow-up continued until December 31, 2018. Prevalent cases were excluded—specifically, those that involved a dementia diagnosis (*ICD-9* 290 or 294; *ICD-10-SE* F00-F04, F051, G30, G31, or A81.0) or receipt of antidementia medication (cholinesterase inhibitors and/or memantine) before the index date.

### Exposures and Outcomes

Participants were categorized by the number of teeth present at the time of dementia diagnosis, with severe tooth loss (STL) defined as <10 remaining teeth and moderate tooth loss (MTL) as 10 to 19 remaining teeth, and compared with an unexposed reference group with ≥20 remaining teeth (Kassebaum et al. 2014). Tooth count constituted natural permanent teeth, with or without restorations, excluding third molars, dental implants, dentures, and bridges. The primary outcome of interest was all-cause mortality. For the secondary outcome, cognitive decline progression was assessed by repeated scores on the Mini Mental State Examination (MMSE), as derived from the SveDem.

### Statistical Analyses

Descriptive statistics were summarized as mean (SD) or median (IQR) for numerical variables and counts (percentages) for categorical variables. Exposure data were obtained from the SKaPa by using all available historical records up to the date of dementia diagnosis, with all exposure variables treated as categorical covariates. Missing data were handled through listwise deletion.

Longitudinal MMSE scores were analyzed according to joint modeling of longitudinal and survival data to account for informative censoring due to death. The longitudinal component was modeled with a linear mixed effects model. The survival component was specified as a flexible parametric survival model that included longitudinal MMSE response as a covariate, along with exposure and adjustment variables. Cox proportional hazards regression was used to estimate hazard ratios (HRs) for mortality rate, and Poisson regression was applied to calculate incidence rates.

Sensitivity analyses for unmeasured confounding were conducted by E-values, summarizing the minimum strength of association that an unmeasured confounder would need to have with the exposure and the outcome to fully explain the observed associations (Ding and Vanderweele 2016; Vanderweele 2017). Potential nonproportional hazards in the Cox models were evaluated by interaction terms with log(time) and log(time)^2^, allowing estimation of time-specific HRs over 1 to 8 y. Absolute risk estimates were derived from restricted mean survival time regression with an identity link, providing differences in restricted mean time lost by tooth count. Finally, sensitivity analyses of longitudinal MMSE changes included additional adjustment for baseline MMSE, restricted to individuals with at least 1 follow-up measurement.

All analyses were performed unadjusted and adjusted on the basis of subject matter knowledge of covariates at the time of dementia diagnosis: age, sex, civil status, income, education, and Charlson Comorbidity Index (Quan et al. 2005). The Charlson Comorbidity Index is a validated method for categorizing comorbidities based on *ICD* diagnosis codes, and it is widely used for adjusting for the burden of systemic diseases. The index covers the 17 most relevant and comorbid conditions, each assigned a weighted score based on the associated risk of mortality (Quan et al. 2005). Missing data were minimal (*n* = 19 across covariates) and were handled through listwise deletion in adjusted analyses.

Statistical tests were 2-tailed and conducted at a 5% significance level. Analyses were performed in SAS/STAT software (version 9.4; SAS Institute Inc.) and R (version 4.4.1; R Core Team). Joint modeling was conducted in the JM package (version 1.5-2), with dependencies survival (version 3.8-3) and nlme (version 3.1-167).

## Results

### Participant Characteristics

**Figure 1.**
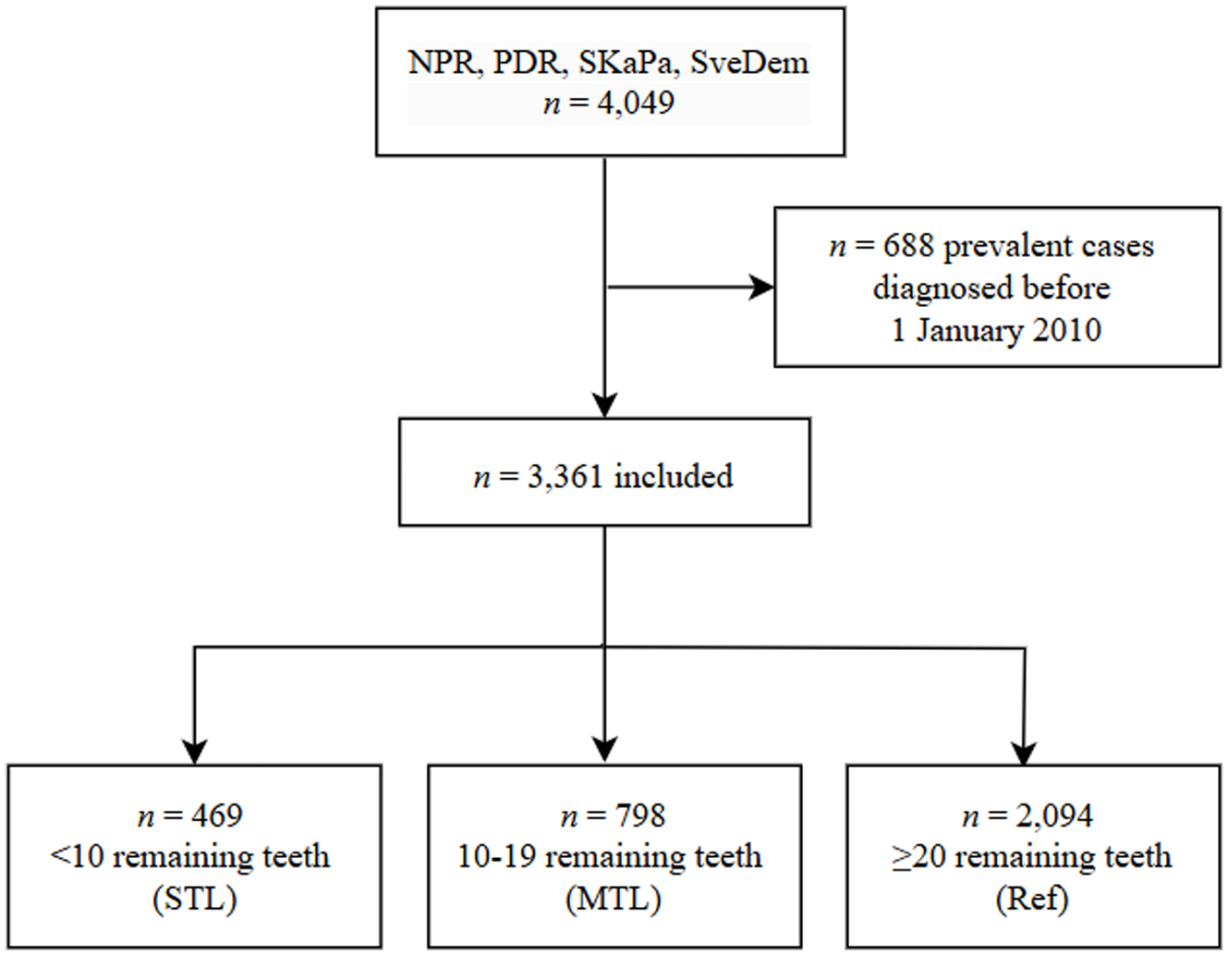
Flowchart illustrating the sample selection process. MTL, moderate tooth loss; NPR, National Patient Register; PDR, National Prescribed Drug Register; Ref, reference; SKaPa, Swedish Quality Registry for Caries and Periodontal diseases; STL, severe tooth loss; SveDem, Swedish Registry for Cognitive/Dementia disorders.

A total of 4,049 dementia cases were identified in the SKaPa and SveDem during the 2010–2013 period. After prevalent cases with a diagnosis before January 1, 2010, were excluded, 3,361 participants remained in the analysis set (Fig. 1) and were followed for a median 5.6 y (IQR, 0.3 to 9.0). Of these, 469 had STL, 798 had MTL, and 2,094 had ≥20 teeth. The mean age at dementia diagnosis was 73.1 y (SD, 6.1), and 54% of participants were female (Table 1). Participants with higher tooth counts (≥20 teeth) generally had higher income and education levels, whereas those with STL had more comorbidities and lower socioeconomic indicators.

**Table 1.** Demographics by Tooth Count Category at Dementia Diagnosis.

	Full Analysis Set (*N* = 3,361)	STL (<10 Teeth) (*n* = 469)	MTL (10 to 19 Teeth) (*n* = 798)	Reference (≥20 Teeth) (*n* = 2,094)
Age, y, mean (SD)	73.1 (6.1)	74.3 (4.9)	74.7 (4.8)	72.3 (6.6)
Female sex	1,826 (54.3)	241 (51.4)	423 (53.0)	1,162 (55.5)
Cohabitation status				
Married	1,886 (56.4)	224 (48.5)	420 (52.9)	1,242 (59.5)
Unmarried	243 (7.3)	39 (8.4)	49 (6.2)	155 (7.4)
Divorced	601 (18.0)	104 (22.5)	158 (19.9)	339 (16.3)
Widowed	611 (18.3)	95 (20.6)	167 (21.0)	349 (16.7)
Income, ×100 SEK, mean (SD)	1,737 (1,665)	1,444 (622)	1,573 (1,171)	1,865 (1,946)
Education				
<9 y	1,122 (33.6)	207 (44.8)	371 (46.7)	544 (26.1)
Primary school	258 (7.7)	35 (7.6)	57 (7.2)	166 (8.0)
Secondary school	1,312 (39.3)	178 (38.5)	272 (34.3)	862 (41.3)
University	535 (16.0)	34 (7.4)	79 (9.9)	422 (20.2)
Charlson Comorbidity Index, mean (SD)	4.3 (2.1)	4.9 (2.2)	4.7 (2.1)	4.1 (2.1)
No. of teeth, median (IQR)	22 (16 to 26)	4 (1 to 7)	16 (14 to 18)	25 (22 to 27)
Dental implants	200 (6.0)	59 (12.6)	83 (10.4)	58 (2.8)

Data are presented as *n* (%) unless noted otherwise.

MTL, moderate tooth loss; STL, severe tooth loss.

### Mortality Rate

**Figure 2.**
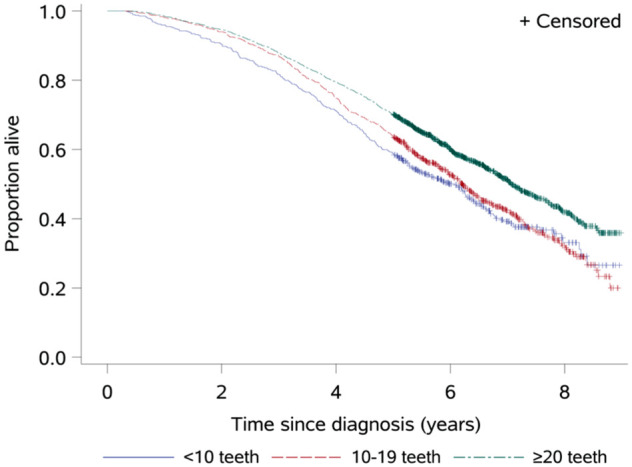
Kaplan-Meier survival curves by number of teeth at the time of dementia diagnosis. *Median survival time for each group is the length of time corresponding to the probability of 0.5.

HRs for mortality in relation to tooth count are presented in Table 2 and Figure 2. Participants with STL and MTL had a higher crude mortality rate than those with ≥20 teeth (HR, 1.40 [95% CI, 1.22 to 1.60] and 1.29 [95% CI, 1.16 to 1.44], respectively), which was not significant after covariate adjustment (adjusted HR, 1.12 [95% CI, 0.97 to 1.28] and 1.08 [95% CI, 0.97 to 1.21]). Sensitivity analyses for unmeasured confounding indicated that a hypothetical confounder associated with tooth loss and mortality by a risk ratio of 1.30 to 1.38 would be required to fully account for the observed point estimates of the HRs. The median survival time was 7.1 y (95% CI, 6.9 to 7.4) for the reference group as compared with 6.1 y (95% CI, 5.3 to 6.4) for the STL group and 6.2 y (95% CI, 5.9 to 6.5) for the MTL group, as shown in Figure 2.

**Table 2.** Risk of All-Cause Mortality by Number of Teeth at Dementia Diagnosis: Events, Incidence Rates, and Hazard Ratios for Severe (<10 Teeth) and Moderate (10 to 19 Teeth) Tooth Loss as Compared with ≥20 Teeth.

No. of Teeth	Events, *n* (%)	IR (95% CI) per 100 Person-Years	Crude HR (95% CI)	*P* Value	Adjusted HR^a^ (95% CI)	*P* Value
<10 (*n* = 469)	266 (56.7)	11.3 (10.1 to 12.8)	1.40 (1.22 to 1.60)	<0.001	1.12 (0.97 to 1.28)	0.12
10 to 19 (*n* = 798)	454 (56.8)	10.7 (9.8 to 11.8)	1.29 (1.16 to 1.44)	<0.001	1.08 (0.97 to 1.21)	0.17
≥20 (*n* = 2,094)	975 (46.6)	8.5 (8.0 to 9.0)	1.00 (Reference)		1.00 (Reference)	

Incidence rates were estimated by Poisson regression with log follow-up time as offset. Crude and adjusted hazard ratios were estimated by Cox proportional hazards regression. Missing covariate data occurred in 7 participants in the severe tooth loss group, 4 in the moderate tooth loss group, and 8 in the reference group; these individuals were excluded from adjusted analyses.

HR, hazard ratio. IR, incidence rate.

a Adjusted for age, sex, civil status, income, education, and Charlson Comorbidity Index.

Additional analyses accounting for time-varying effects revealed evidence of nonproportional hazards in the unadjusted comparison between the STL group and those with ≥20 teeth (*P* = 0.022). The crude HR declined over time from 2.09 (95% CI, 1.52 to 2.86) at 1 y to 1.27 (1.08 to 1.49) at 5 y and was nonsignificant at 7 y (HR, 1.25; 95% CI, 0.95 to 1.65). In contrast, no evidence of time-varying effects was observed in the adjusted models (all *P* > 0.25; Appendix Table 1 and Fig. 1). Analyses of restricted mean survival time indicated increasing absolute survival differences over time. When compared with individuals with ≥20 teeth, those in the STL group had an estimated 0.3 y (~3 mo) of life lost at 5 y (95% CI, 0.19 to 0.54) and 0.6 y (~7 mo) lost at 8 y (95% CI, 0.36 to 0.84). These differences were attenuated and no longer statistically significant after adjustment for confounding variables (Appendix Table 2 and Fig. 2).

### Longitudinal Changes in MMSE

Changes in MMSE scores by number of teeth are presented in Table 3. An annual decline in crude MMSE scores was observed across all groups: −1.26 units (95% CI, −1.43 to −1.09) in STL, −1.50 units (95% CI, −1.65 to −1.34) in MTL, and −1.42 units (95% CI, −1.52 to −1.33) in the reference group. Adjusted mean differences in decline showed no statistically significant association: 0.10 units (95% CI, −0.15 to 0.35) for STL and −0.01 units (95% CI, −0.20 to 0.17) for MTL as compared with the reference group. A sensitivity analysis additionally adjusting for baseline MMSE scores yielded similarly null results (Appendix Table 3).

**Table 3.** Longitudinal Changes in Mini Mental State Examination Scores by Number of Teeth at Dementia Diagnosis.

No. of Teeth	Annual Change (95% CI)	Unadjusted		Adjusted^a^	
		Difference (95% CI) vs. ≥20 Teeth	*P* Value	Difference (95% CI) vs. ≥20 Teeth	*P* Value
<10	−1.26 (−1.43 to −1.09)	0.16 (−0.03 to 0.36)	0.10	0.10 (−0.15 to 0.35)	0.43
10–19	−1.50 (−1.65 to −1.34)	−0.07 (−0.26 to 0.11)	0.42	−0.01 (−0.20 to 0.17)	0.90
≥20	−1.42 (−1.52 to −1.33)	0.00 (Reference)		0.00 (Reference)	

Statistical analyses were conducted via joint modeling of longitudinal and survival data, accounting for informative censoring due to death. Missing covariate data occurred in 7 participants in the severe tooth loss group (<10 teeth), 4 in the moderate tooth loss group (10 to 19 teeth), and 8 in the reference group (≥20 teeth); these individuals were excluded from adjusted analyses.

a Adjusted for age, sex, civil status, income, education, and Charlson Comorbidity Index.

## Discussion

We explored tooth loss as a prognostic indicator for dementia, with the hypothesis of increased mortality risk as well as increased progression of cognitive decline among individuals with dementia. The first hypothesis was not confirmed, as associations were not statistically significant after adjustment for potential confounding factors. If an association is borne out with further studies covering a longer follow-up period, some potential explanations can be suggested. Systemic implications of tooth loss have been discussed in a neurodegenerative context—for example, those mainly attributed to the effects of mastication impairment, including declining somatosensory output to the brain, nutritional deficiency, and forced changes in food textures (Kato et al. 1997; Yamamoto and Hirayama 2001; Tsutsui et al. 2007; Okamoto et al. 2019).

Our second hypothesis was not fulfilled as all groups experienced a similar decline in MMSE scores. One could speculate that the exposed groups had increased mortality rates, shortening the length of time over which MMSE scores could be observed. Indeed, the individuals with STL and MTL had a median survival time that was 1 y shorter than that of the reference group. To address this issue, we conducted joint modeling analysis to account for informative censoring due to death, which showed that our results seemed to hold.

Increasing evidence points to an association of compromised oral health with the development of dementia (Chen et al. 2017; Lee et al. 2020; Ma et al. 2022). For instance, in monozygotic twins discordant for dementia, tooth loss before age 35 y was identified as the only significant risk factor for Alzheimer disease (multivariate odds ratio, 1.68; 95% CI, 1.21 to 2.32; Gatz et al. 2006). A randomized controlled trial revealed improvements in cognitive performance among individuals with mild Alzheimer disease through a 6-mo oral health intervention, although the short follow-up period may not have been sufficient to observe the full effects of the intervention (Chen et al. 2022). To our knowledge, the current study is the first to investigate tooth loss as a factor for mortality and progression risk in persons with dementia. Our findings hint at an impact of tooth loss in established dementia, particularly with STL, but further work with a longer follow-up is warranted.

Observational research requires appropriate study design and proper confounding factor control to provide reliable and meaningful insights, particularly for interdisease associations. A major strength of this study is the use of national health and quality assurance registries, which enabled us to capitalize on comprehensive longitudinal data in following dementia onset, severity, and other outcomes in relationship with tooth loss. Moreover, access to registry data allowed for thorough adjustment of important sociodemographic and clinical covariates. By using secondary data from these registries, we could eliminate observer bias. All available data in the SKaPa are provided in electronic dental charts by clinicians primarily for the sake of their clinical work and secondarily for research purposes. A validation study of diagnostic accuracy of caries between the electronic dental charts and corresponding data retrieved from the SKaPa revealed satisfactory reliability (Mensah et al. 2021).

Some limitations that could have influenced outcomes and interpretation should be noted, including an absence of information about cigarette smoking, which represents a potential confounder. Additionally, data about dentures were limited. The verification of regular denture use remains challenging, which could introduce uncertainty in the interpretation of its potential impact. Furthermore, the SKaPa lacks information of clinical attachment loss, a key variable for periodontal diseases, representing a limitation and preventing periodontal disease classification according to the current classification system of the European Federation of Periodontology and the American Academy of Periodontology (Caton et al. 2018). In addition, these limitations may cause residual confounding, restricting the interpretability. Thus, tooth loss was the primary focus for this study, as a surrogate marker of cumulative dental disease experience and compromised oral health. Another limitation is the follow-up period, which may not have been sufficient to tease out the associations of factors with these complex conditions, possibly explaining the inconsistent findings for dementia progression, likely affecting the inference. This study did not account for potential informative censoring due to mediating factors such as depression, which could be associated with poorer outcomes and a higher likelihood of loss to follow-up. If individuals with more severe depressive symptoms were less likely to attend follow-up and they had worse prognoses, this may have biased the observed trend estimates upward in the more morbid groups.

A common issue in epidemiologic research is reverse causality, which in our case would entail the effects of dementia on oral health instead of the reverse. Some evidence suggests that oral health and dental care use decline among people with dementia (Fereshtehnejad et al. 2018). This decline could be attributed to limitations on daily activities and an increased dependency on caregivers or family members. Dementia sometimes involves behavioral changes that can lead to individuals showing reduced cooperation and to challenges in maintaining proper oral hygiene routines. By focusing on persons newly diagnosed with dementia, we likely reduced the risk of reverse causality. We also excluded people with a dementia diagnosis or antidementia medication prescription before the index date for the same reason. Other sources of potential bias should be acknowledged, such as index event bias, which may affect associations by conditioning on individuals who lived long enough to be diagnosed with dementia. Further studies with richer covariate data—including additional risk factors for dementia and mortality, such as smoking and APOE ε4 status—are warranted to better account for residual confounding. We adjusted for socioeconomic status by using civil status, income, and education, which may not fully capture the influence of socioeconomic factors on the exposure-outcome relationship. Although these variables are correlated, such correlations do not bias the estimated associations of interest but may increase uncertainty around individual covariate estimates.

With an expected worldwide increase in dementia cases, these findings may be generalizable to the international public health setting. By using data from nationwide registries (SveDem, National Prescribed Drug Register, National Patient Register), we successfully included a large proportion of the available population within the age interval of greater dementia prevalence. Our study population had a mean age of 73 y (SD, 6) and median follow-up >5 y. This is consistent with the SveDem data, which show that 19% of individuals are diagnosed between the ages of 65 and 74 y and 48% between 75 and 84 y (SveDem 2019).

The Swedish government recently presented dental care as a key part of the new national dementia strategy, highlighting the importance of acknowledging oral health throughout dementia progression (Government of Sweden 2025). Preceding this addition, research in a large Swedish population had shown a stable decline in dental care visits among people who had received a dementia diagnosis (Fereshtehnejad et al. 2018). This finding highlights the need for continued efforts to incorporate regular contact with dental care services for this vulnerable group.

In conclusion, tooth loss was not independently associated with increased mortality among persons newly diagnosed with dementia following covariate adjustment. No association was found between tooth loss and cognitive decline over time. As oral health is modifiable and often overlooked in dementia care, further studies are warranted to clarify its role in prognosis and to explore underlying mechanisms, such as nutritional status, systemic inflammation, and access to dental care.

## Supplemental Material

sj-docx-1-jdr-10.1177_00220345251384633 – Supplemental material for Tooth Loss in Individuals with Dementia: A Swedish Register-Based Cohort Study Supplemental material, sj-docx-1-jdr-10.1177_00220345251384633 for Tooth Loss in Individuals with Dementia: A Swedish Register-Based Cohort Study by M. Mohammadi, J. Holmer, H. Imberg, H. Albrektsson, M. Eriksdotter and K. Buhlin in Journal of Dental Research

## Author Contributions

M. Mohammadi, contributed to conception and design, data acquisition, analysis, and interpretation, drafted and critically revised the manuscript; J. Holmer, M. Eriksdotter, contributed to conception and design, data acquisition and interpretation, critically revised the manuscript; H. Imberg, H. Albrektsson, contributed to design, data analysis and interpretation, critically revised the manuscript; K. Buhlin, contributed to conception and design, data acquisition, analysis, and interpretation, critically revised the manuscript. All authors gave final approval and agreed to be accountable for all aspects of the work.
