# Supplementary material for: Tooth Loss in Individuals with Dementia: A Swedish Register-Based Cohort Study
Source: J Dent Res. 2025 Oct 22;105(1):149–55. doi: 10.1177/00220345251384633 (PMC12701894; doi:10.1177/00220345251384633)
Supplement: Supplementary file 1 [file sj-docx-1-jdr-10.1177_00220345251384633.docx]

This supplement has been provided by the authors to offer readers additional information about their work.

Supplement to

**Tooth Loss in Individuals with Dementia: A Swedish Register-based Cohort Study**

Authored by

**Mehrad Mohammadi^1^, Jacob Holmer^1, 2^, Henrik Imberg^3, 4^, Henrik Albrektsson^4^, Maria Eriksdotter^5, 6^, Kåre Buhlin^1, 7^**

^1^Division of Periodontology, Department of Dental Medicine, Karolinska Institutet, Huddinge, Sweden

^2^Department of Periodontology, Specialist Dental Clinic, Västmanland Hospital Västerås, Region Västmanland, Västerås, Sweden

^3^Department of Molecular and Clinical Medicine, Institute of Medicine, Sahlgrenska Academy, University of Gothenburg, Gothenburg, Sweden

^4^Statistiska Konsultgruppen Sweden, Gothenburg, Sweden

^5^Division of Clinical Geriatrics, Centre for Alzheimer Research, Department of Neurobiology, Care Sciences and Society, Karolinska Institutet, Stockholm, Sweden

^6^Inflammation and Aging Theme, Karolinska University Hospital, Stockholm, Sweden

^7^Department of Oral and Maxillofacial Diseases, University of Helsinki, Helsinki, Finland

**Contents**

**Supplemental Table S1.** Time-dependent hazard ratios for all-cause mortality by tooth count at dementia diagnosis…………………………………………………………………………..3

**Supplemental Table S2.** Restricted mean time lost (RMTL) and group differences in years lost by tooth count at dementia diagnosis...................................................................................4

**Supplemental Table S3.** Differences in annual MMSE change by number of teeth at dementia diagnosis, adjusted for baseline MMSE……………………………………………..5

**Supplemental Figure S1.** Time-dependent hazard ratios for all-cause mortality by tooth count at dementia diagnosis…………………………………………………………………....6

**Supplemental Figure S2.** Restricted mean time lost (RMTL) over 8 years of follow-up by tooth count at dementia diagnosis……………………………………………………………...7

**Supplemental Table S1.** Time-varying hazard ratios for all-cause mortality by tooth count at the time of dementia diagnosis.

| Follow-up time (years) | HR (95% CI)  <10 vs ≥20 teeth | HR (95% CI)  10–19 vs ≥20 teeth | aHR* (95% CI)  <10 vs ≥20 teeth | aHR* (95% CI)  10–19 vs ≥20 teeth |
| --- | --- | --- | --- | --- |
| 1 | 2.09 (1.52, 2.86) | 1.15 (0.83, 1.60) | 1.47 (1.00, 2.16) | 0.91 (0.62, 1.34) |
| 2 | 1.54 (1.23, 1.92) | 1.18 (0.97, 1.43) | 1.25 (1.00, 1.57) | 0.97 (0.80, 1.18) |
| 3 | 1.37 (1.14, 1.65) | 1.23 (1.05, 1.44) | 1.15 (0.95, 1.40) | 1.03 (0.87, 1.21) |
| 4 | 1.30 (1.12, 1.52) | 1.28 (1.12, 1.46) | 1.09 (0.93, 1.28) | 1.08 (0.94, 1.23) |
| 5 | 1.27 (1.08, 1.49) | 1.33 (1.18, 1.51) | 1.04 (0.88, 1.23) | 1.12 (0.99, 1.28) |
| 6 | 1.26 (1.02, 1.54) | 1.38 (1.18, 1.62) | 1.01 (0.81, 1.24) | 1.17 (1.00, 1.37) |
| 7 | 1.25 (0.95, 1.65) | 1.43 (1.16, 1.76) | 0.98 (0.74, 1.30) | 1.21 (0.98, 1.50) |
| 8 | 1.26 (0.89, 1.78) | 1.48 (1.13, 1.94) | 0.96 (0.66, 1.38) | 1.25 (0.95, 1.65) |
| Statistical analyses were conducted using Cox regression including interaction terms for group comparisons (<10 vs ≥20 teeth and 10–19 vs ≥20 teeth) with log(time) and log(time)², to account for potential non-proportional hazards over time.  Hazard ratios (HRs) represent time-specific estimates at 1 through 8 years after dementia diagnosis.  Wald tests for non-proportional hazards, assessed via linear contrasts of log(time) and log(time)² interaction terms by group, yielded *P*=0.02 for <10 vs ≥20 teeth and *P*=0.50 for 10–19 vs ≥20 teeth in unadjusted models, and *P*=0.26 and *P*=0.37, respectively, in adjusted analyses.  Missing covariate data occurred in 7 participants in the STL group, 4 in the MTL group, and 8 in the Reference group; these individuals were excluded from adjusted analyses.  *Adjusted for age, sex, civil status, income, education, and Charlson Comorbidity Index at dementia diagnosis.  **Abbreviations**: aHR, adjusted hazard ratio; CI, confidence interval; HR, hazard ratio. | | | | |

**Supplemental Table S2.** Restricted mean time lost (RMTL) and differences in years lost by tooth count at dementia diagnosis.

| Follow-up time (years) | Time lost (95% CI), years | | | RMTL difference (95% CI) | | Adjusted RMTL difference* (95% CI) | |
| --- | --- | --- | --- | --- | --- | --- | --- |
|  | <10 teeth | 10–19 teeth | ≥20 teeth (ref) | <10 vs ≥20 teeth | 10–19 vs ≥20 teeth | <10 vs ≥20 teeth | 10–19 vs ≥20 teeth |
| 1 | 0.01 (0.01, 0.02) | 0.01 (0.00, 0.01) | 0.00 (0.00, 0.01) | −0.01 (−0.01, −0.00) | −0.00 (−0.01, 0.00) | −0.00 (−0.01, 0.00) | 0.00 (−0.00, 0.00) |
| 2 | 0.08 (0.06, 0.11) | 0.04 (0.03, 0.06) | 0.04 (0.03, 0.05) | −0.04 (−0.07, −0.02) | −0.01 (−0.02, 0.01) | −0.01 (−0.04, 0.01) | 0.01 (−0.01, 0.02) |
| 3 | 0.22 (0.17, 0.28) | 0.14 (0.11, 0.17) | 0.12 (0.11, 0.14) | −0.10 (−0.16, −0.05) | −0.02 (−0.05, 0.02) | −0.03 (−0.08, 0.02) | 0.02 (−0.01, 0.05) |
| 4 | 0.46 (0.38, 0.54) | 0.33 (0.28, 0.38) | 0.29 (0.26, 0.32) | −0.17 (−0.26, −0.09) | −0.04 (−0.10, 0.02) | −0.05 (−0.13, 0.03) | 0.03 (−0.03, 0.09) |
| 5 | 0.82 (0.70, 0.93) | 0.64 (0.56, 0.71) | 0.54 (0.49, 0.58) | −0.28 (−0.40, −0.15) | −0.10 (−0.19, −0.01) | −0.09 (−0.20, 0.03) | 0.02 (−0.06, 0.11) |
| 6 | 1.28 (1.13, 1.43) | 1.06 (0.96, 1.16) | 0.89 (0.82, 0.95) | −0.39 (−0.55, −0.23) | −0.17 (−0.29, −0.05) | −0.13 (−0.28, 0.03) | 0.01 (−0.11, 0.12) |
| 7 | 1.83 (1.65, 2.02) | 1.59 (1.46, 1.72) | 1.33 (1.25, 1.41) | −0.51 (−0.71, −0.31) | −0.26 (−0.41, −0.11) | −0.17 (−0.36, 0.02) | −0.02 (−0.17, 0.12) |
| 8 | 2.46 (2.24, 2.68) | 2.22 (2.07, 2.38) | 1.87 (1.77, 1.96) | −0.60 (−0.84, −0.36) | −0.36 (−0.54, −0.18) | −0.19 (−0.42, 0.04) | −0.06 (−0.23, 0.12) |
| Statistical analyses were conducted using restricted mean survival time (RMST) regression with an identity link to estimate years of life lost and group differences at 1 through 8 years after dementia diagnosis. Restricted mean time lost (RMTL) was calculated as the follow-up duration minus the RMST.  Missing covariate data occurred in 7 participants in the STL group, 4 in the MTL group, and 8 in the Reference group; these individuals were excluded from adjusted analyses.  *Adjusted for age, sex, civil status, income, education, and Charlson Comorbidity Index at dementia diagnosis. **Abbreviations:** CI, confidence interval; ref, reference; RMST, restricted mean survival time. | | | | | | | |

**Supplemental Table S3.** Differences in annual MMSE change by number of teeth at dementia diagnosis, adjusted for baseline MMSE values.

|  | Difference in annual MMSE change (95% CI)  <10 vs ≥20 teeth | *P* | Difference in annual MMSE change (95% CI)  10–19 vs ≥20 teeth | *P* |
| --- | --- | --- | --- | --- |
| Adjusted Model 1* | 0.10 (-0.13, 0.34) | 0.40 | 0.02 (-0.29, 0.34) | 0.88 |
| Adjusted Model 2† | 0.03 (-0.22, 0.28) | 0.80 | -0.13 (-0.47, 0.20) | 0.43 |
| Statistical analyses were conducted using joint modelling of longitudinal and survival data to account for informative censoring due to death.  Values are presented as mean differences in annual change in MMSE score between tooth count groups, with 95% confidence intervals (CIs).  * Model 1: Adjusted for baseline MMSE score. † Model 2: Additionally adjusted for age, sex, civil status, income, education, and Charlson Comorbidity Index.  **Abbreviations:** CI, confidence interval; MMSE, mini-mental state examination. | | | | |

**Supplemental Figure S1.** Time-varying hazard ratios for all-cause mortality by tooth count at dementia diagnosis. Estimates were derived from Cox regression models with interaction terms for group comparisons (<10 vs ≥20 teeth and 10–19 vs ≥20 teeth) and log(time) and log(time)² to account for non-proportional hazards. Shaded areas represent 95% confidence intervals. Unadjusted (left) and adjusted for age, sex, civil status, income, education, and Charlson Comorbidity Index (right). Evidence of time-varying effects was observed for the <10 vs ≥20 teeth comparison in unadjusted models (*P*=0.02), but not for 10–19 vs ≥20 teeth or in any of the adjusted comparisons (all *P*>0.25).

**Supplemental Figure S2.** Restricted mean time lost (RMTL) over 8 years of follow-up by tooth count at dementia diagnosis. Estimates were derived from restricted mean survival time regression using an identity link to model time lost due to death. Lines represent <10 teeth (blue solid line), 10–19 teeth (red dashed line), and ≥20 teeth (green long-dash–short-dash line). Shaded areas indicate 95% confidence intervals.
